# The acidic domain of the hepatitis C virus NS4A protein is required for viral assembly and envelopment through interactions with the viral E1 glycoprotein

**DOI:** 10.1371/journal.ppat.1007163

**Published:** 2019-02-07

**Authors:** Allison E. Roder, Christine Vazquez, Stacy M. Horner

**Affiliations:** 1 Department of Molecular Genetics & Microbiology, Duke University Medical Center, Durham, NC, United States of America; 2 Department of Medicine, Duke University Medical Center, Durham, NC, United States of America; The University of Chicago, UNITED STATES

## Abstract

Hepatitis C virus (HCV) assembly and envelopment are coordinated by a complex protein interaction network that includes most of the viral structural and nonstructural proteins. While the nonstructural protein 4A (NS4A) is known to be important for viral particle production, the specific function of NS4A in this process is not well understood. We performed mutagenesis of the C-terminal acidic domain of NS4A and found that mutation of several of these amino acids prevented the formation of the viral envelope, and therefore the production of infectious virions, without affecting viral RNA replication. In an overexpression system, we found that NS4A interacted with several viral proteins known to coordinate envelopment, including the viral E1 glycoprotein. One of the NS4A C-terminal mutations, Y45F, disrupted the interaction of NS4A with E1. Specifically, NS4A interacted with the first hydrophobic region of E1, a region previously described as regulating viral particle production. Indeed, we found that an E1 mutation in this region, D72A, also disrupted the interaction of NS4A with E1. Supernatants from HCV NS4A Y45F transfected cells had significantly reduced levels of HCV RNA, however they contained equivalent levels of Core protein. Interestingly, the Core protein secreted from these cells formed high order oligomers with a density matching the infectious virus secreted from wild-type cells. These results suggest that this Y45F mutation in NS4A causes secretion of low-density Core particles lacking genomic HCV RNA. These results corroborate previous findings showing that the E1 D72A mutation also causes secretion of Core complexes lacking genomic HCV RNA, and therefore suggest that the interaction between NS4A and E1 is involved in the incorporation of viral RNA into infectious HCV particles. Our findings define a new role for NS4A in the HCV lifecycle and help elucidate the protein interactions necessary for production of infectious virus.

## Author summary

RNA viruses, which encompass both established and emerging pathogens, pose significant public health challenges. Viruses in the family *Flaviviridae*, including Dengue virus, Zika virus, and hepatitis C virus (HCV), continue to cause morbidity and mortality worldwide. One HCV protein, NS4A, acts in several steps of the viral lifecycle; however, how it contributes to viral particle production is not understood. Here, we investigated the role of one region of NS4A, the C-terminal acidic domain, in regulating the viral lifecycle. We found that amino acids within this domain are important for viral envelopment and the production infectious particles, specifically through interaction with the E1 glycoprotein. NS4A interacts with the first hydrophobic domain of E1. Disruption of this interaction, by either mutation of E1 or NS4A, prevents the production of infectious virus particles and instead results in release of low-density Core protein complexes that lack HCV RNA into the cellular supernatant. Overall, our results reveal that NS4A is important for late stages of the HCV lifecycle and suggest that the interaction between NS4A and E1 may regulate the incorporation of viral RNA into the virion for the formation of infectious HCV particles.

## Introduction

Hepatitis C virus (HCV) is a positive-sense RNA virus of the genus *Hepacivirus* in the *Flaviviridae* family. Over 70 million people worldwide are chronically infected with HCV and this chronic infection can lead to liver cirrhosis and hepatocellular cancer [1]. In the years spanning 2003–2013, HCV-related deaths numbered more than any other CDC-reported infectious disease [2]. Despite the availability of newly designed, highly effective direct-acting antivirals, disease prevalence remains high, and no vaccine exists for the virus [3–5].

HCV encodes a single stranded, positive-sense RNA genome of approximately 9.6 kilobases in length. Upon virus entry into hepatocytes, the viral genome is translated to form a single polyprotein. The polyprotein is co- and post-translationally cleaved by both host and viral proteases, including the NS3-NS4A viral protein complex, to form ten individual proteins. These ten proteins include both structural proteins, which eventually make up the virion, and nonstructural proteins, which coordinate RNA replication and the other steps in the viral lifecycle, including virion assembly and envelopment (reviewed in [6]).

The late stages of the viral lifecycle, including assembly and envelopment, are just beginning to be dissected. While many details of these processes are not understood, recent work has uncovered several key steps that lead to production of infectious virus. Following RNA replication, HCV RNA is shuttled to the lipid droplet where Core protein accumulates, oligomerizes, and also recruits the NS3 and NS5A proteins [7–10]. NS5A is thought to play a role in RNA recruitment to the lipid droplet, whereas NS3 likely aids in movement of Core bound to RNA from the lipid droplet to nearby sites on the endoplasmic reticulum (ER) [11–16]. This process is coordinated by the NS2 protein which acts as a bridge between the nonstructural protein NS3 and the structural protein E2, to link virion assembly at the lipid droplet to envelopment at the ER [17–22]. The role of NS2 in these steps is supported by the actions of the p7 protein [23, 24]. Lastly, Core oligomers bound to viral RNA bud into the ER lumen, acquiring an ER-derived lipid bilayer envelope that contains the viral E1 and E2 transmembrane glycoproteins [25, 26]. It is unclear what signals are necessary for the membrane curvature that results in budding, but it is clear that E1 and E2 are necessary for successful envelopment, as deletion of E1 and E2 prevents formation of the viral envelope and production of infectious virions [24]. Following virion budding into the ER lumen, the virion is transported through the very-low-density lipoprotein (VLDL) secretory pathway acquiring apolipoproteins and other lipids, and is ultimately released from the cell in a noncytolytic manner as a lipoviroparticle [27–30]. In addition to the viral proteins mentioned here, a number of host proteins also facilitate HCV morphogenesis [31](reviewed in [32]).

In addition to its roles in viral assembly and envelopment, the NS3-NS4A protein complex has several other well-established functions in the HCV lifecycle. It is essential for viral polyprotein processing, viral RNA replication, and negative regulation of antiviral innate immunity (reviewed in [33]). NS3 functions as both a serine protease and an RNA helicase and requires its cofactor NS4A to enhance these activities and to target the protease complex to intracellular membranes [34, 35]. NS4A is 54 amino acids long and contains three domains, an N-terminal transmembrane domain that anchors NS3 to intracellular membranes, a central NS3-interaction domain required for proper folding of NS3, and a C-terminal domain that contains a kink region followed by an acidic region with a high number of acidic amino acids [36–39]. While the specific roles of NS4A in the HCV lifecycle are largely thought to occur indirectly through its function as a cofactor for NS3, mutation-based studies of the NS4A acidic domain suggest some independent roles for NS4A in regulating the HCV lifecycle, including during assembly and envelopment, as described below [36, 40].

There is strong evidence that NS3 and NS4A are each involved in the steps of virion assembly and envelopment. Specifically, NS3 has been shown to be involved in viral particle production through interactions with both Core and NS2 [15, 21, 22, 41]. In addition, culture adaptive amino acid mutations in the α_0_ helix of NS3 have been shown to promote viral assembly [16]. Separately, HCV particle production is also regulated by specific amino acids in the NS4A acidic domain such that when mutated, infectious virus formation is inhibited without affecting RNA replication. Some of these mutations can be partially rescued by compensatory amino acid substitutions in NS3, suggesting that NS3 and NS4A together can cooperate to regulate HCV particle production [40]. However, both the extent of the role of NS4A in assembly and envelopment and the specific function of NS4A in regulating the production of infectious HCV remain unclear.

Here, we define a new role for the NS4A protein in regulating HCV envelopment. We have identified amino acids in the acidic domain of NS4A that are required for the formation of the viral envelope. Further, we have found that NS4A alone can interact with a number of viral proteins that coordinate viral envelopment, including Core, E1, E2, and NS5A. Interestingly, disruption of the NS4A-E1 interaction, by mutation of either NS4A or E1, prevents envelopment of the HCV particle and results in secretion of Core particles that are not associated with viral RNA. Taken together, our findings reveal a new role for NS4A in coordinating the HCV lifecycle and define new viral interactions that lead to successful HCV particle envelopment.

## Results

### A Y45F mutation in the hepatitis C virus NS4A protein causes a decrease in infectious viral titer

**Fig 1:**
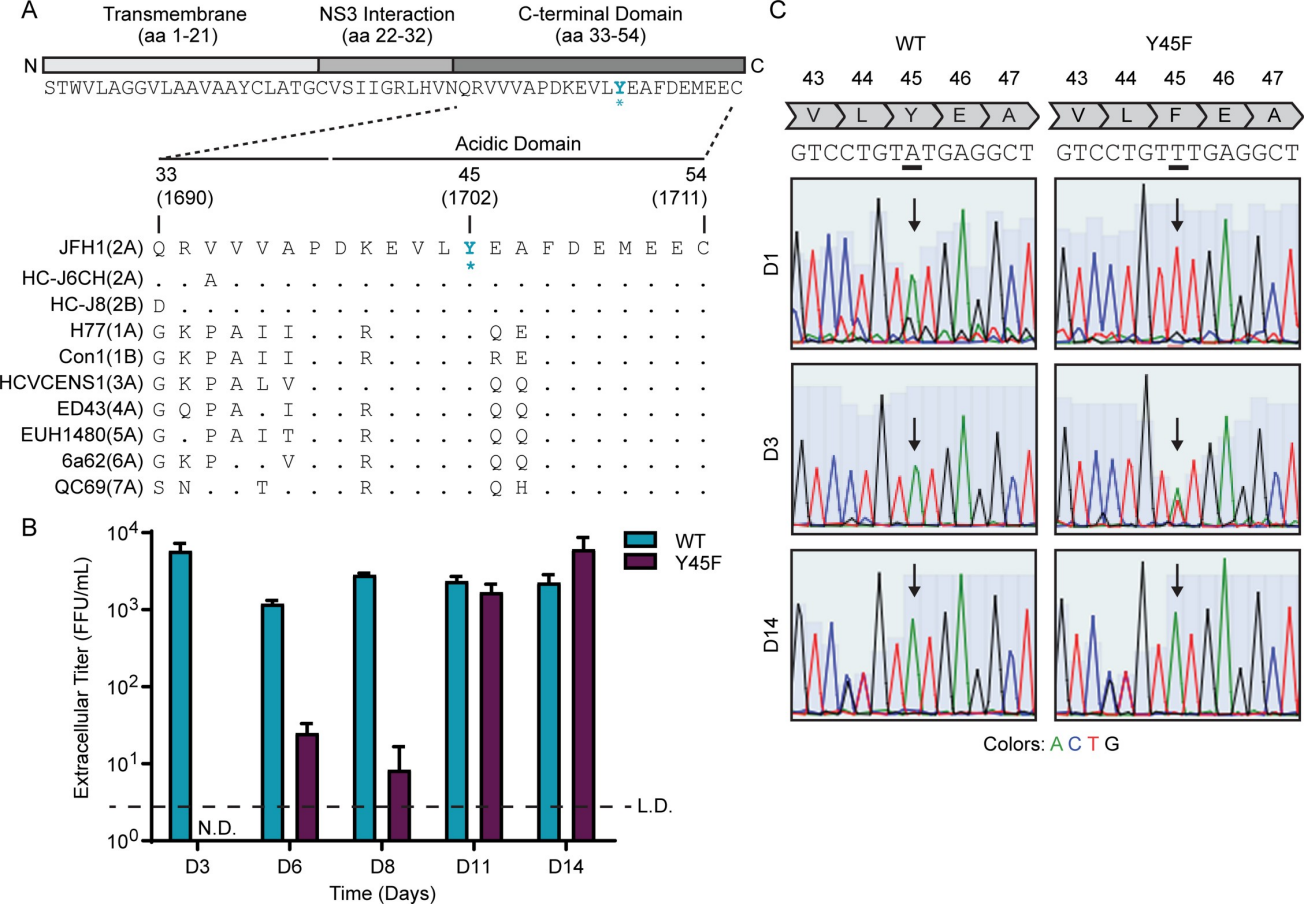
A Y45F mutation in hepatitis C virus NS4A causes a decrease in infectious viral titer. (A) Schematic of the NS4A protein. * indicates location of Y45F mutation. Numbers correspond with the amino acid position within NS4A (aa 1–54) or the full-length polyprotein (aa 1690–1711). Strain names are listed as found in the Los Alamos HCV sequence database. (B) Focus forming assay of supernatants harvested from Huh7.5 cells at the indicated days post-electroporation with HCV WT or NS4A Y45F *in vitro* transcribed RNA. FFU/mL = focus forming units/milliliter. Values are presented as mean ± SD (n = 3). Data are representative of three independent experiments. N.D. = not detected. L.D. = limit of detection. (C) Sequencing of the NS4A region (nt 4710–5251) of cDNA amplified from Huh7.5 cells transfected with HCV WT or NS4A Y45F RNA (JFH1) in (B) at indicated days.

The acidic domain of NS4A has significant sequence homology between all HCV genotypes, with amino acids 40–54 in the acidic domain differing by at most 3 amino acids (Fig 1A). In particular, the tyrosine residue at position 45 is conserved across 99.5% of the 865 HCV genome sequences in the Los Alamos HCV sequence database, which includes sequences from seven genotypes [42]. While previous studies have implicated the acidic domain of NS4A in regulating HCV RNA replication and particle production, the mechanism of this regulation was not explored [40]. We sought to investigate how the NS4A acidic domain contributes to HCV particle production. We engineered a structurally conservative amino acid substitution, changing the tyrosine residue (Y, TAT) at position 45 to a phenylalanine (F, TTT) in a genotype 2A strain of HCV, Japanese fulminant hepatitis-1 (JFH1) [43]. We then generated wild-type (WT) or NS4A Y45F *in vitro* transcribed RNA and transfected it into Huh7.5 cells. At 3-days post-transfection, while the WT RNA produced more than 3 logs of infectious virus, no infectious virus was detected from cells transfected with RNA containing the Y45F mutation, as measured by focus forming assay (Fig 1B). After several cell passages, Y45F RNA began to produce infectious virus, and after 14 days it produced equivalent titers to that of the WT virus (Fig 1B). Sequencing of the NS4A region of HCV RNA extracted from cells at 1, 3, and 14 days post-transfection revealed that the Y45F mutation had reverted back to WT by day 14, with some reversion detected as early as day 3 (Fig 1C). The entire HCV genome was also sequenced following passage of the Y45F-transfected cells over 6 weeks. In this time, we only observed reversion of the Y45F mutation back to WT and did not detect the emergence of any second-site mutations. These results reveal that substitution of the tyrosine at position 45 with phenylalanine in NS4A prevents production of infectious HCV, indicating that Tyr-45 is required for the production of infectious virus.

### The NS4A Y45F mutation in HCV does not alter viral RNA replication

**Fig 2:**
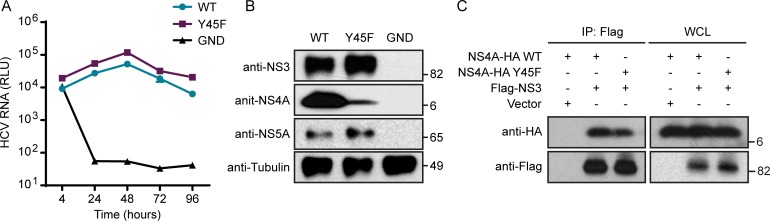
The NS4A Y45F mutation in HCV does not alter viral RNA replication. (A) Renilla luciferase assay to measure HCV replicon luciferase reporter (JFH1-SGR-luc) activity from Huh7.5 cells at indicated times following electroporation. GND = lethal mutation in HCV NS5B RNA-dependent RNA polymerase. RLU = *Renilla* luciferase units. Data are presented as mean ± SEM (n = 3). (B) Immunoblot analysis of extracts of Huh7.5 cells at 72 hours post-transfection with indicated HCV RNA. (C) Immunoblot analysis of anti-Flag immunoprecipitated extracts and whole cell lysates (WCL) from Huh7.5 cells transfected with indicated tagged HCV proteins or vector.

To determine if the loss of infectious HCV production by the NS4A Y45F amino acid change was due to altered HCV RNA replication, we engineered the Y45F mutation into an HCV subgenomic replicon construct containing a luciferase reporter and measured luciferase production over time following transfection of Huh7.5 cells with *in vitro* transcribed HCV RNA. We found that HCV replicon RNA with the Y45F mutation in NS4A replicated as efficiently as WT, while the HCV RNA with a lethal mutation in the NS5B RNA dependent RNA polymerase (GND) did not replicate (Fig 2A). Additionally, the HCV proteins NS3, NS4A and NS5A were expressed in lysates harvested at 48 hours post-transfection of either WT or Y45F RNA, indicating that the Y45F mutation did not affect the production of these viral proteins (Fig 2B). Of note, the epitope of the NS4A antibody is in the C-terminal domain of NS4A that contains Tyr-45. Therefore, the reduced detection of the NS4A band by immunoblotting in the mutant condition suggests that the Y45F mutation reduces NS4A recognition by this antibody (Fig 2B). Further, the fact that HCV RNA replication is not altered by the Y45F mutation indicates that the NS4A protein must be stably expressed, as NS4A is required for HCV RNA replication [36, 40, 44]. We also confirmed that the Y45F mutation did not impact RNA replication in another genotype of HCV by measuring long-term HCV RNA replication in cells transduced with a genotype 1B subgenomic replicon RNA encoding a G418 selectable marker [45]. Indeed, there was no difference in the number of G418-resistant colonies that arose between WT and Y45F transduced cells, revealing that the Y45F mutation also did not impact replication of a genotype 1B subgenomic replicon (S1A and S1B Fig). Since the interaction of NS3 with NS4A is essential for viral replication, we tested if the Y45F mutation affected this interaction using a co-immunoprecipitation experiment with overexpressed proteins. The results show that the Y45F mutation does not alter NS3-NS4A complex formation (Fig 2C). Together, these results indicate that the Y45F mutation in NS4A does not alter HCV RNA replication, HCV protein expression, or NS3-NS4A complex formation. Therefore, the NS4A Y45F mutation in HCV must cause a defect at a later stage of the viral lifecycle.

### The NS4A Y45F mutation inhibits viral envelopment

**Fig 3:**
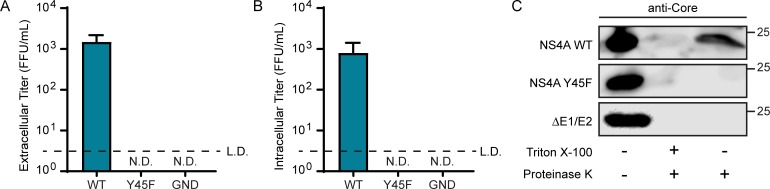
The NS4A Y45F mutation inhibits HCV envelopment. (A, B) Focus forming assay of supernatants for extracellular titer, or cellular lysates for intracellular titer, respectively, from Huh7.5 cells at 48 hours post-electroporation with HCV WT, Y45F, or GND (JFH1) *in vitro* transcribed RNA. N.D. = not detected. L.D. = limit of detection. (C) Immunoblot analysis for HCV Core protein of cell lysates of Huh7.5 cells at 48 hours post-electroporation with *in vitro* transcribed HCV RNA subjected to the indicated treatments in a proteinase K protection assay. ΔE1/E2 = complete deletion of E1 and E2 coding regions. For panels A and B, data are presented as mean ± SEM (n = 3). C is representative of 3 independent experiments.

As the NS4A Y45F mutation did not alter HCV RNA replication but did prevent infectious virus production, we next tested if this mutation affected viral assembly and envelopment or viral release. We first examined if the Y45F mutation caused a viral release defect by measuring both intracellular and extracellular titer. We transfected Huh7.5 cells with WT, Y45F, or GND HCV RNA and measured the viral titer from the supernatant (extracellular titer) or from lysates generated by freeze-thaw cycles (intracellular titer) by using a focus forming assay. As before, HCV NS4A Y45F RNA did not produce extracellular titer (Fig 3A), and here we found that it also did not produce intracellular titer (Fig 3B). Taken together, these results indicate that the Y45F mutation impairs viral particle production prior to the formation of fully infectious virions.

An infectious HCV virion contains viral RNA, encapsidated by the viral Core protein, surrounded by an outer lipid envelope containing the viral glycoproteins, E1 and E2, and cellular lipids and lipoproteins [32, 46, 47]. Taking advantage of these structural properties of the HCV virion, we next tested if the Y45F mutation prevented viral envelopment by using a proteinase K protection assay. The HCV Core protein in enveloped virions is protected from degradation following proteinase K treatment by the outer lipid envelope [24]. Because the HCV glycoproteins are required for acquisition of the lipid bilayer membrane, a viral RNA with a deletion in the E1 and E2 coding region (ΔE1/E2) can be used a negative control for envelopment [24]. Lysates were harvested from HCV RNA (WT, Y45F, or ΔE1/E2) transfected Huh7.5 cells, incubated with proteinase K, and analyzed by immunoblot for Core. We found that while Core was protected from proteinase K digestion in WT, it was not protected in lysates containing the Y45F mutation, similar to ΔE1/E2 (Fig 3C). These data indicate that the Y45F mutation prevents envelopment of the virion, resulting in a lack of both intracellular and extracellular viral titer, suggesting that Y45 may be an important residue for HCV envelopment.

### Multiple amino acids in the acidic domain of NS4A are required for HCV envelopment

**Fig 4:**
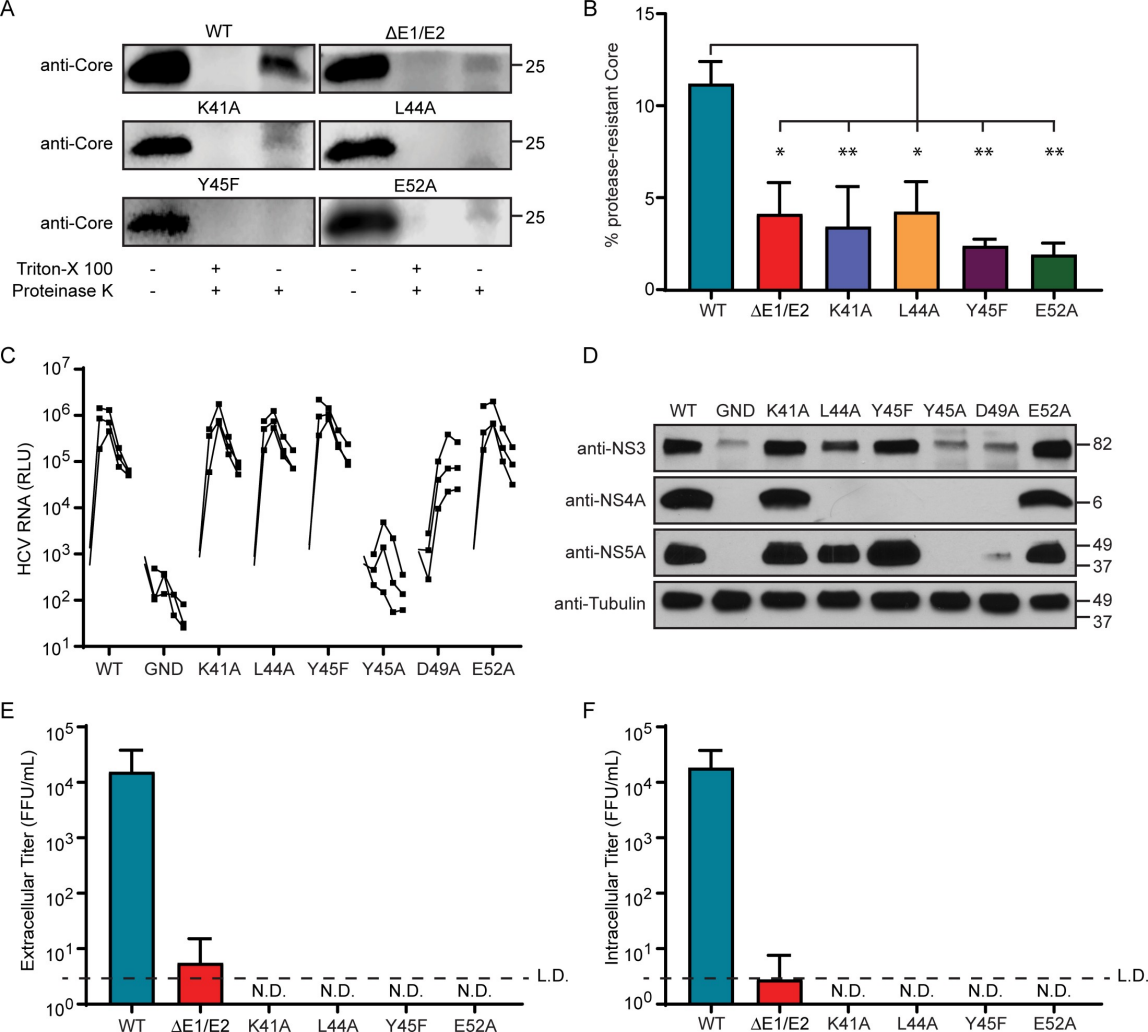
Multiple amino acids in the acidic domain of NS4A are required for HCV envelopment. (A) Immunoblot analysis of HCV Core protein from lysates of Huh7.5 cells at 48 hours post-electroporation of *in vitro* transcribed HCV RNA (JFH1) containing the indicated mutations in the NS4A region, after the indicated treatments in a proteinase K protection assay. (B) Quantification of percentage of protease-resistant Core relative to untreated Core from (A). ΔE1/E2 = deletion in the E1 and E2 coding regions. Data are presented as mean ± SEM (n = 3) and were analyzed by one-way ANOVA. *P < 0.05, **P < 0.01. (C) *Renilla* luciferase assay to measure HCV replicon luciferase reporter (JFH1-SGR-luc) activity from Huh7.5 cells following electroporation of indicated constructs, with individual replicates from three experiments plotted. GND = lethal mutation in HCV NS5B RNA-dependent RNA polymerase. RLU = *Renilla* luciferase units. (D) Immunoblot analysis of lysates from the 72-hour time point of (C). (E, F) Focus forming assay of supernatants for extracellular titer or cellular lysates for intracellular titer, respectively, from Huh7.5 cells at 48 hours post-electroporation with *in vitro* transcribed HCV RNA (JFH1) containing the indicated mutations in NS4A. N.D. = not detected. L.D. = limit of detection. For (B, E, F), data are presented as mean ± SEM (n = 3). Panel A is representative of three independent experiments.

Based on our findings that HCV RNA with the NS4A Y45F mutation has a defect in viral envelopment, we hypothesized that other amino acids in the NS4A C-terminal acidic domain may also play a role in envelopment. To test this, we introduced several mutations into the acidic domain of NS4A that were previously found to be important for production of infectious HCV particles (K41A, L44A, and E52A) and tested their effects on viral envelopment [40]. We performed a proteinase K protection assay, as in Fig 3, and found that the K41A, L44A and E52A mutants all resulted in a quantifiable decrease in protease-resistant Core as compared to WT, suggesting that these mutations also caused a defect in envelope formation (Fig 4A and 4B).

We additionally tested the impact of these amino acids on RNA replication, HCV protein expression, and production of both intracellular and extracellular titer; and also tested two additional mutations with known replication defects, Y45A and D49A, as controls [40]. HCV RNA containing the NS4A K41A, L44A, and E52A mutations all replicated and expressed HCV proteins to a similar extent as WT, while NS4A D49A and Y45A showed mild to severe replication defects (Fig 4C and 4D). All of these mutations prevented intracellular and extracellular infectious virus from being produced, as seen previously by others (Fig 4E and 4F) [40]. Taken together, these data show that multiple amino acids within the acidic domain of NS4A are important for formation of the viral envelope and production of infectious virus.

### NS4A Y45 is required for NS4A interaction with the E1 glycoprotein

**Fig 5:**
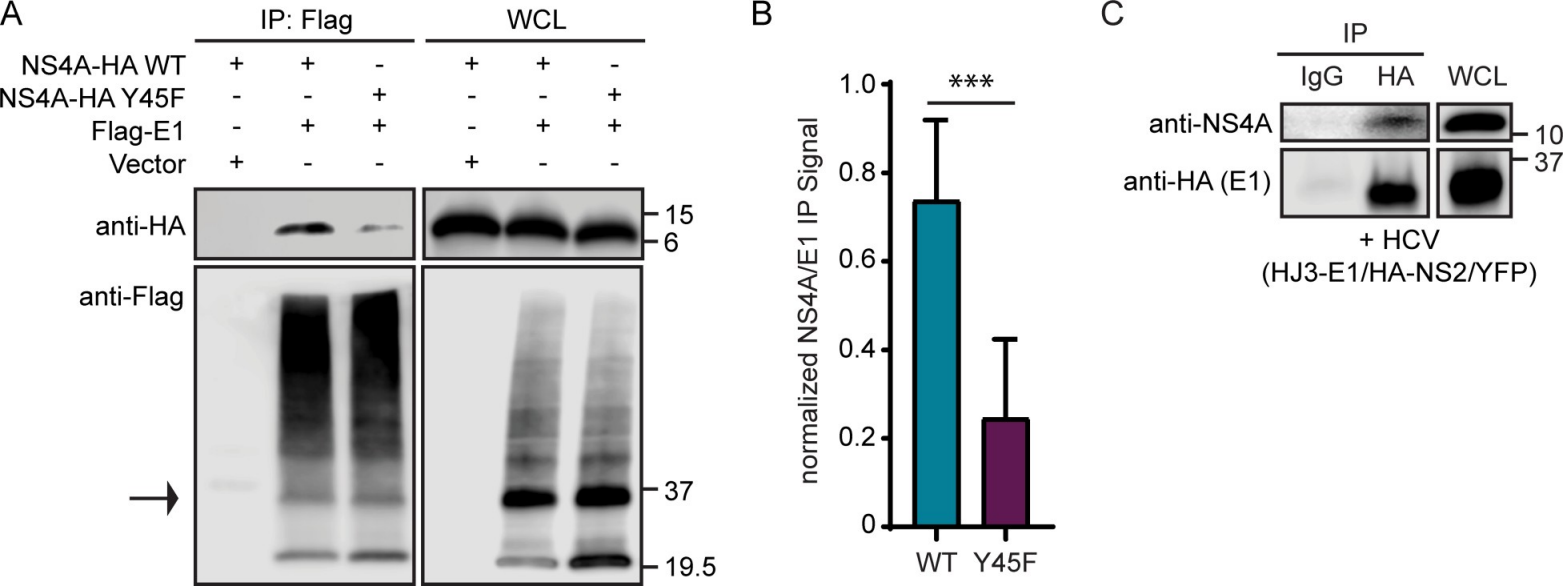
NS4A Y45 is required for NS4A interaction with the E1 glycoprotein. (A) Immunoblot analysis of anti-Flag immunoprecipitated extracts from Huh7.5 cells transfected with indicated HCV proteins or vector. (B) Quantification of the signal of NS4A relative to E1 from the immunoprecipitation from (A). Data are presented as mean ± SEM (n = 3) and were analyzed by unpaired t-test. ***P< 0.001. (C) Immunoblot analysis of anti-HA or anti-IgG immunoprecipitated extracts from Huh7.5 cells at 48 hours post-electroporation with *in vitro* transcribed HCV HJ3-E1/HA-NS2/YFP [41] RNA which contains an N-terminal HA tag on E1. Panel C is representative of three independent experiments.

Because a complex network of HCV proteins regulates HCV assembly and envelopment, we hypothesized that NS4A may facilitate an interaction between either structural (Core, E1, or E2) or nonstructural (p7, NS2 or NS5A) proteins to regulate these processes. Therefore, we first tested if overexpressed NS4A WT or Y45F interacted with Core, E1, or E2 using co-immunoprecipitation in Huh7.5 cells. We found that overexpressed NS4A WT interacts with Core, E1 and E2 (Figs 5A, S2A and S2B). While Core and E2 interactions with NS4A were equivalent for WT and Y45F (S2A and S2B Fig), the NS4A Y45F mutation greatly decreased NS4A and E1 interaction (Fig 5A and 5B). We also performed this immunoprecipitation with NS4A and E1 overexpression constructs from two additional genotypes, 1B and 3. Indeed, we found that in both of these genotypes, NS4A interacts with E1 during overexpression and that the Y45F mutation reduces this interaction (S1C and S1D Fig). To determine if NS4A WT interacts with E1 in the context of HCV infection we transfected Huh7.5 cells with an infectious clone of HCV containing an N-terminal HA tag on E1 [41]. We then immunoprecipitated E1 by using an anti-HA antibody and found that, indeed, NS4A and E1 can interact during HCV infection (Fig 5C). We also tested the interactions of NS4A with p7, NS2, and NS5A, nonstructural proteins that all have roles in HCV envelopment [10–12, 17–21, 23, 24]. NS4A did interact with NS5A, but this interaction was not altered by the Y45F mutation in NS4A (S2D Fig). We found no interaction between overexpressed NS4A WT and either NS2 or p7 (S2C Fig). To determine if a tyrosine at position 45 was absolutely essential for NS4A-E1 interaction, we created several additional mutations at position 45 (Y45T, Y45R, and Y45D) and tested their ability to interact with E1. Interestingly, both NS4A Y45T and Y45D, but not Y45R, interacted with E1 (S3 Fig). Together, these data show that NS4A can bind to Core, E1, E2, and NS5A, and that specific mutation of NS4A at Y45 can disrupt its binding to the E1 protein.

### NS4A binds to the first hydrophobic region of E1

**Fig 6:**
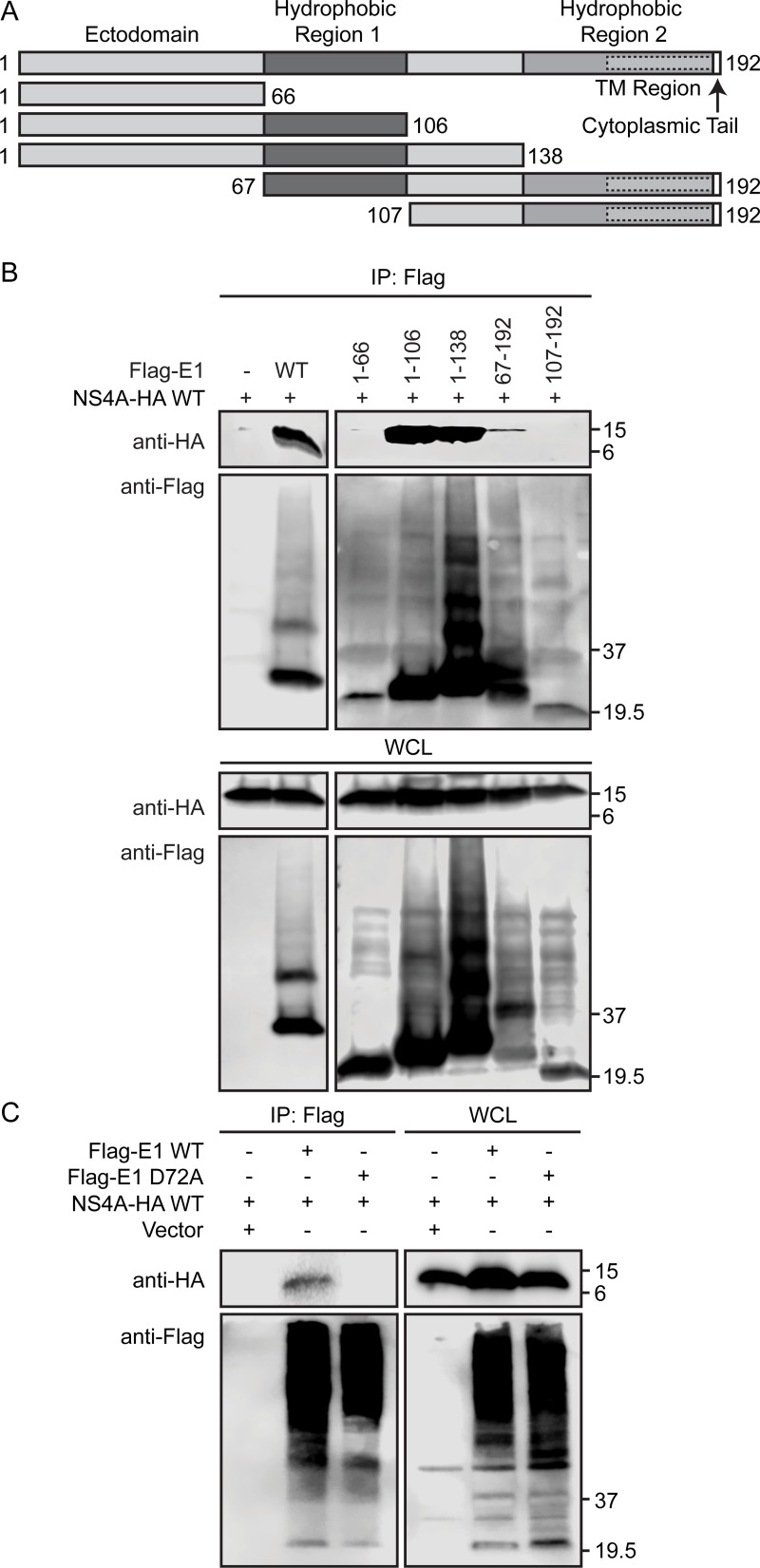
NS4A binds the first hydrophobic region of E1. (A) Schematic of the HCV E1 protein with functional domains indicated and amino acids numbered based on the E1 coding region of JFH1. (B) Immunoblot analysis of anti-Flag immunoprecipitated extracts and whole cell lysate (WCL) from Huh7.5 cells transfected with NS4A-HA WT and Flag-tagged E1, either full-length (WT) or as indicated. Data is representative of 3 independent experiments. (C) Immunoblot analysis of anti-Flag immunoprecipitated extracts and whole cell lysate (WCL) from Huh7.5 cells transfected with Flag-tagged E1 WT or D72A and NS4A-HA or vector. Panel C is representative of 2 independent experiments.

To investigate the mechanism of how the NS4A-E1 interaction might facilitate viral envelopment, we mapped the binding site of NS4A on E1. The E1 and E2 glycoproteins are translated in the ER membrane and are cleaved from the viral polyprotein by a host protease. After cleavage, E1 and E2 form a stable heterodimer and are retained in the ER. E1 has an N-terminal ectodomain, two hydrophobic regions, and a transmembrane region near the C-terminus (Fig 6A) (reviewed in [48]). We therefore created a series of N-terminally Flag-tagged E1 truncation mutants based on these known domains of E1 (Fig 6A). We overexpressed the truncation mutants with NS4A containing a C-terminal HA-tag in Huh7.5 cells and then performed Flag immunoprecipitations followed by immunoblotting for NS4A-HA. We found that NS4A co-immunoprecipitated with E1 aa 1–106, aa 1–138, and aa 67–192, all of which contain the first hydrophobic region of E1, but did not interact with aa 1–66 or aa 107–192, which lack this region (Fig 6B). These data suggest that NS4A binds to E1 via the first hydrophobic region of the protein. The first hydrophobic region of E1 has previously been implicated in HCV particle production, and several amino acids within this region are important for viral infectivity [49, 50]. In particular, one specific E1 mutation, D72A (HCV polyprotein aa 263), attenuated viral infectivity even though Core protein was still secreted into cellular supernatants [50]. Because of these data and our finding that NS4A binds to the first hydrophobic region of E1, which contains D72, we hypothesized that the D72A mutation in E1 would specifically disrupt the NS4A-E1 interaction, similar to the Y45F mutation in NS4A (Fig 5A). Indeed, the E1 D72A mutation diminished the interaction between NS4A and E1 during overexpression, supporting the conclusion that NS4A binds to the first hydrophobic region of E1 (Fig 6C). Together, these data suggest that NS4A binds to the first hydrophobic region of E1 and this interaction specifically involves NS4A Y45 and E1 D72.

### NS4A Y45F results in release of Core oligomers devoid of HCV RNA

**Fig 7:**
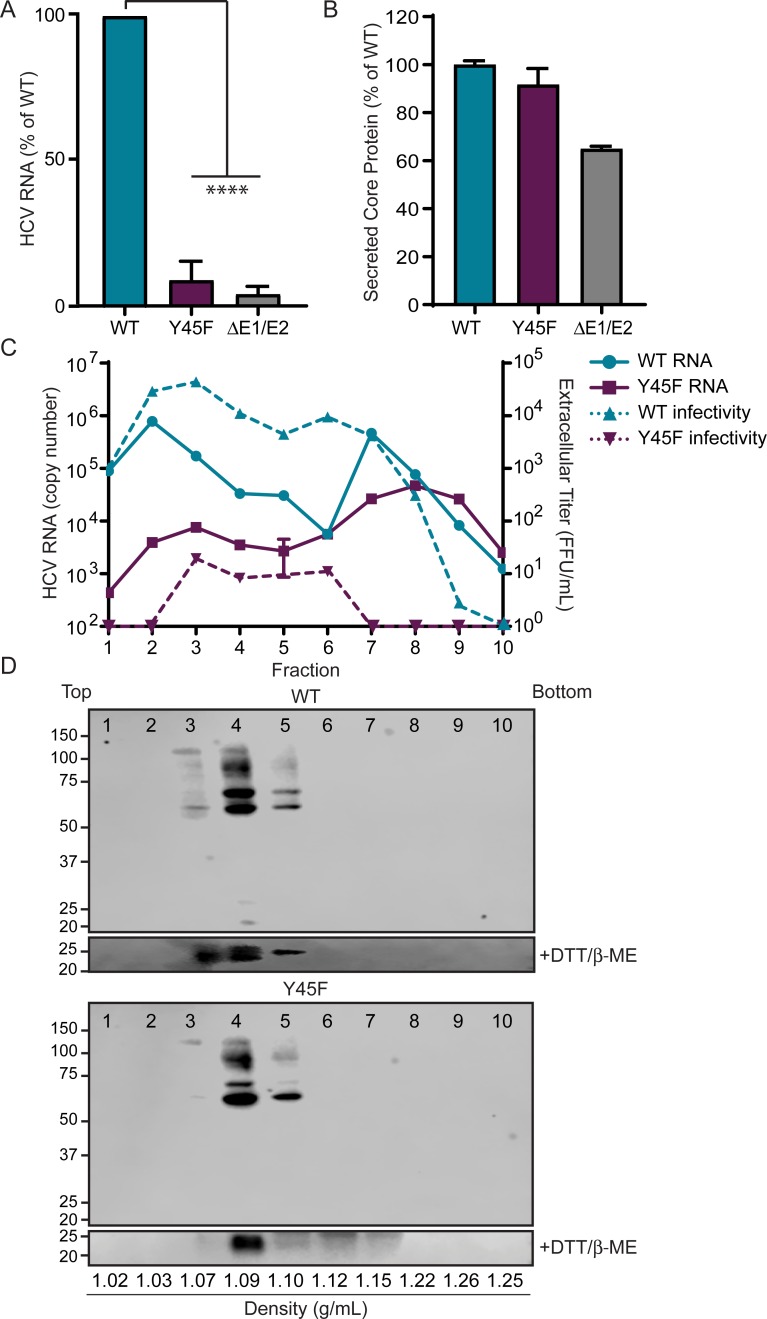
The NS4A Y45F mutation results in production of partially formed virions. Supernatants harvested from Huh7.5 cells 48 hours post-electroporation of NS4A WT, NS4A Y45F, or ΔE1/E2 *in vitro* transcribed HCV RNA (JFH1) were analyzed for HCV RNA by RT-qPCR (A) or Core protein by ELISA (B). Data in A are presented as mean ± SEM (n = 3) and analyzed by unpaired t-test. ****P<0.0001. Data in B are representative of 2 independent experiments and are presented as mean ± SD (n = 2). Supernatant harvested from Huh7.5 cells 48 hours post-electroporation with *in vitro* transcribed HCV WT or NS4A Y45F RNA (JFH1) were concentrated, fractionated over a 10–50% iodixanol gradient, and collected into 10 equal fractions. Fractions were analyzed by focus-forming assay for infectivity, represented as FFU/mL, and RT-qPCR for HCV RNA, shown as a measure of copy number (C) and by immunoblot for Core (D). In the bottom panels of the immunoblots, fractions were treated with 2-mercaptoethanol and DTT and then boiled before immunoblotting for anti-Core. Fractions 1–10 correspond with fractions running from top to bottom of the gradient, with the densities listed at the bottom. Data in C is presented as mean ± SD (n = 3); C-D are representative of 2 independent experiments.

Others have shown that the D72A mutation in E1 in full-length HCV RNA results in release of non-infectious, partially formed virions containing Core protein but lacking HCV RNA [50]. Taken together with our findings that NS4A binds to the first hydrophobic region of E1, which contains D72, we hypothesized that Y45F may have a similar phenotype. Therefore, we measured the secretion of HCV RNA and Core protein into supernatants from cells replicating HCV NS4A WT or Y45F RNA. As a control, we also measured secretion of RNA and Core protein from cells transfected with HCV ΔE1/E2 RNA. We found that the Y45F mutation resulted in lower levels of extracellular HCV RNA as compared to WT, similar to ΔE1/E2, as measured by RT-qPCR. However, secretion of Core into the supernatant was only slightly reduced, as measured by ELISA (Fig 7A and 7B). In contrast, Core levels in the supernatant from HCV ΔE1/E2 were about 65% of what was seen for WT. These results for Y45F were similar to the previously published profile of Core and RNA secreted from cells transfected with the D72A mutant [50]. Because of these data, we sought to profile the viral components in the supernatants from Y45F-transfected cells. We collected and concentrated cellular supernatants from HCV NS4A WT or Y45F-transfected cells and then centrifuged these samples over iodixanol gradients. We collected 10 equal fractions from the top, with fraction 1 having the lowest density and fraction 10 having the highest density. Each fraction was analyzed by RT-qPCR for HCV RNA, by focus forming assay for viral titer, and by immunoblot for Core protein. In the WT samples, fractions 2 and 3 had the highest levels of both HCV RNA and viral titer (Fig 7C). These fractions also contained high molecular weight complexes of Core protein (Fig 7D, lanes 3–5). These Core complexes could be reduced to the molecular weight of monomeric Core protein (21 kDa) by treating the fractions with reducing agents and boiling them before SDS-PAGE analysis (Fig 7D, bottom panels). We observed a second peak of HCV RNA in fractions 7, 8, and 9, however these fractions had significantly less viral titer (Fig 7D, lanes 7–9). Therefore, the HCV RNA in these higher density fractions is likely non-infectious and may represent secreted membrane-associated RNA from replication complexes [51]. On the other hand, we saw little to no infectious viral titer from any fraction in the Y45F samples and found the majority of HCV RNA present in fractions 7–9, while the expected infectious fractions (2–4) had little RNA (Fig 7C). Interestingly, high molecular weight complexes of Core protein were still observed in fractions 3–5, similar to the distribution of Core in WT samples (Fig 7D). The fact that Core formed oligomers that were in a different fraction than the peak of HCV RNA suggests that the Y45F mutation results in release of partially formed virions containing Core protein oligomers but lacking HCV RNA. Furthermore, the fraction containing the majority of Core protein in both WT and Y45F also contained the E1 and E2 glycoproteins, suggesting that the primary component missing from the partially formed virions in Y45F is HCV RNA (S4B Fig).

Aside from Y45, we identified several amino acids within the acidic domain of NS4A that have roles in HCV envelopment (Fig 4A and 4B). To determine if one of these amino acids regulates the same function during envelopment as Y45, we also performed the fractionation on supernatant from cells transfected with HCV RNA containing the NS4A K41A mutation. Similar to what we observed earlier, neither Y45F or K41A RNA produced infectious virions (Figs 4E and S4A). We also included the E1 D72A mutant in these experiments to confirm the work of others showing that E1 D72A causes a defect in viral envelopment (S4A Fig) [50]. While the profile of RNA and Core protein from the supernatants of cells transfected with E1 D72A RNA was similar to that of cells transfected with the NS4A Y45F RNA, we found that the distribution of the K41A RNA in the supernatant was different from that of both NS4A Y45F and E1 D72A. The RNA secreted from the K41A transfected cells was present in fractions 2–4 as in WT, but not in fractions 7–9, where the peak of RNA for Y45F and D72A was found. However, the supernatant of cells transfected with K41A RNA did contain high molecular weight oligomers of Core protein in the infectious fraction, similar to that seen with WT and Y45F (S4A and S4B Fig). These results suggest that while K41A does have a defect in envelopment, K41 likely contributes to envelopment through a different mechanism than Y45 and E1 D72. Together our data suggest that the NS4A Y45F mutation results in secretion of low-density, partially formed virions lacking HCV RNA.

## Discussion

Our results define a new role for NS4A in the late stages of the HCV lifecycle. Specifically, we have found that the acidic domain of NS4A is important for regulating assembly and that mutation of specific amino acids within this domain prevents formation of the viral envelope. Further, we have identified new interactions between NS4A and HCV structural and nonstructural viral proteins. This suggests that NS4A may act to bridge two stages of the HCV lifecycle, linking virion assembly at the lipid droplet to virion envelopment at the ER, similar to the actions of the NS2 protein. Importantly, we found that NS4A binds to E1 and that antagonizing this interaction with one amino acid change in NS4A prevents viral envelopment. We mapped the binding site of NS4A on E1 and found that it interacts with the first hydrophobic region, specifically at D72, an amino acid that is known to be important for viral particle production [49, 50]. Finally, we found that the Y45F mutation in NS4A, which prevents envelopment, also results in secretion of noninfectious, incompletely formed virions that are composed of low-density Core protein oligomers that lack HCV RNA. Together our results reveal a new role for NS4A in coordinating the proper assembly and envelopment of HCV particles to make infectious virus.

The NS4A protein contains only 54 amino acids and yet has three distinct domains with specific functions in the HCV lifecycle. While the functions of the NS4A transmembrane domain and the NS3 interaction domain are largely defined [33], much less is known about the function of the C-terminal region, which contains a high number of acidic amino acids (Fig 1A). We found that mutation of several amino acids in the acidic domain, including Y45F, did not affect viral RNA replication, but did disrupt the formation of the viral envelope and therefore prevented production of infectious virus (Figs 2A, 3A, 3C and 4). Indeed, the presence of a Tyr at amino acid 45 of NS4A was so essential for the viral lifecycle that a viral RNA containing the Y45F mutation reverted back to the WT sequence after only a few days of passage in cell culture (Fig 1B and 1C). Given that a Tyr to Phe change is a structurally conservative mutation removing only the hydroxyl group, it is formally possible that NS4A could be phosphorylated at this tyrosine. Indeed, a Y45D substitution, which mimics the charge of phosphorylation, retained NS4A binding to E1 (S3A Fig). However, it is unlikely that lack of phosphorylation of this tyrosine would be the sole contributor to the envelopment defects, as several other amino acids within this region also prevented envelopment (Fig 4) [40]. Therefore, the acidic domain likely regulates envelopment through the concerted actions of the amino acids in this acidic region of NS4A.

Changing the amino acids in NS4A at K41, L44, Y45, and E52 to alanine all resulted in loss of viral titer due to defects in envelopment (Fig 4). The acidic domain of NS4A, which has been proposed to have an alpha helical structure, is important for replication, and indeed the Y45A change results in less replication [36, 40]. However, because mutation of the other amino acids within this C-terminal domain did not alter viral RNA replication, it is unlikely that they disrupt the conformation of the alpha helix. Structural predictions of this alpha helix suggest that K41, Y45, and E52 all are on one face of the helix while L44 and D49 would be on the opposite face [36]. Therefore, it is possible that the amino acids we studied in this region could facilitate different protein interactions on opposite faces of the protein, each contributing to HCV envelopment. In support of this hypothesis, previous work has shown that an adaptive mutation in NS3 partially rescues an assembly defect resulting from the K41A mutation, suggesting that NS4A can cooperate with NS3 via K41 for viral particle production [40]. We found that NS4A WT and Y45F bound NS3 equivalently (Fig 2C) but that the Y45F mutation prevented NS4A interaction with E1 (Fig 5A). Additionally, analysis of cellular supernatants from cells transfected with HCV NS4A K41A RNA showed a profile of RNA secretion in supernatant fractions that was different than the RNA secreted from both WT and Y45F transfected cells. These data reveal that while the K41A and Y45F mutations both prevent HCV envelopment, these amino acids may function to facilitate different NS4A-protein interactions that regulate envelopment.

Supporting the hypothesis that NS4A interacts with several HCV proteins to coordinate virion envelopment, we did identify several previously unknown interactions of NS4A with overexpressed structural and nonstructural proteins, such as Core, E1, E2, and NS5A. However, while NS2 is thought to be a main organizer of envelopment, we found that NS4A did not interact with NS2 during overexpression. Therefore, these data suggest that during infection NS2 and NS4A likely work together through a multi-protein complex or perform similar roles in the lifecycle [19]. Indeed, NS2 is considered to be the main organizer of envelopment, binding both structural and nonstructural proteins to link viral assembly steps at the lipid droplet to viral envelopment steps at the ER [17–21, 23, 24]. NS4A also binds to proteins involved in both early (Core, NS3, and NS5A) and later (E1 and E2) steps of assembly and envelopment, which could suggest that NS4A may also serve as a link between virion production steps at the lipid droplet and the ER, similar to NS2. Overall, these results suggest that NS2 and NS4A could play similar roles in organizing and facilitating viral envelopment.

We found that NS4A binds to E1 and that this interaction is disrupted by the Y45F mutation in NS4A and also by the D72A mutation in E1 (Fig 5, Fig 6C), suggesting that the NS4A-E1 interaction is important for envelopment of the virion. The E1 protein has an N-terminal ectodomain, two internal hydrophobic domains, a transmembrane domain, and a very short, 2 amino acid cytoplasmic, C-terminal tail (Fig 6A) [48]. The first hydrophobic domain of E1 has previously been shown to bind to Core, and mutations within this region diminish viral particle production [49, 50, 52–54]. Surprisingly, we found that NS4A binds to this region of E1 (Fig 6B). Supporting this finding, we also found that a point mutation in the first hydrophobic domain of E1, D72A, disrupted the NS4A-E1 interaction. Curiously, this E1 domain is hydrophobic and is proposed to associate with the lipid membrane bilayer, while the NS4A acidic domain is cytoplasmic and not known to be membrane-associated. It is possible that the acidic domain of NS4A could associate with the ER membrane to interact with this region of E1. However, it is equally likely that NS4A and E1 can interact through a membrane-associated host protein or with cellular lipids. Future studies designed to determine how NS4A interacts with E1 would yield further insights into the HCV envelopment process.

The finding that NS4A binds to the first hydrophobic region of E1 is particularly interesting, as this region in E1 has recently been shown to regulate viral particle production [49, 50]. In fact, the D72A mutation in E1 (polyprotein aa 263), resulted in decreased viral titer and secretion of Core particles that were devoid of HCV genomic RNA. Further, others have shown that this mutation disrupts the localization of E1 with HCV RNA in fluorescence *in situ* hybridization experiments [50]. In our studies, fractionation of supernatant from cells replicating HCV NS4A Y45F RNA revealed that low-density fractions contained little to no HCV RNA, similar to E1 D72A (Fig 7, S4A Fig). However, these low-density fractions contained secreted Core oligomers, suggesting that these oligomers were associated with cellular lipids or apolipoproteins. Indeed, transfected Core protein has been shown to self-assemble into higher order complexes, and non-enveloped particles have been found in the serum of HCV infected patients [55, 56]. Transfection of Core alone can also alter VLDL secretion, and therefore it is possible that secreted Core may be associated with cellular lipids and lipoproteins [57]. Taken together, these data suggest that the NS4A acidic domain and the E1 first hydrophobic domain cooperate during envelopment to aid in the incorporation of viral RNA into the virion. While NS4A itself does not have RNA binding capability, it does form a complex with NS3, which contains an RNA binding helicase domain, and thus, NS3-NS4A together could cooperate with E1 to incorporate HCV RNA into the developing virion.

Our study contributes new insights into the steps required for HCV to form infectious viral particles. As the viral particle lifecycle stages that occur in association with lipid droplets and the ER are tightly linked and likely occur nearly simultaneously, it’s unclear if nucleocapsid intermediates (Core protein assembled around HCV RNA) exist separate from fully enveloped nucleocapsids [32]. Our data show that Core protein assembles into oligomers prior to envelopment and suggest that the function of NS4A in viral assembly and envelopment is after this Core oligomerization step. Further, the fact that we identified Core protein oligomers that did not contain a protective envelope or HCV RNA suggests that RNA incorporation into the virion at or near envelopment sites could be a necessary signal for virion budding events to occur. Our data therefore support a model by which NS4A interacts with E1 to link viral RNA to Core oligomers in the forming virion and signal the envelopment of the Core-RNA complex.

## Materials and methods

### Cell lines and culture conditions

Huh7.5 cells (gift of Dr. Michael Gale), which have been previously described [45], were maintained in Dulbecco’s modification of eagle’s medium (DMEM; Mediatech) supplemented with 10% fetal bovine serum (FBS; HyClone) and 25 mM HEPES (Thermo-Fisher) at 37°C with 5% CO_2_. The identity of the Huh7.5 cells used in this study was verified by using the Promega GenePrint STR kit (DNA Analysis Facility, Duke University), and cells were verified as mycoplasma free by the LookOut Mycoplasma PCR detection kit (Sigma).

### Plasmids and site-directed mutagenesis

These plasmids have been described previously: psJFH1-p7+NS [58], pHCV-HP WT [45], HJ3-E1/HA-NS2/YFP ([41], gift of Dr. MinKyung Yi), and pFL-J6/JFH-1-FlagE2 ([59], gift of Dr. Matthew Evans). psJFH1-p7+NS is a culture adapted strain of JFH1 containing 7 mutations within p7 and the nonstructural proteins that we have described previously [58]. pJFH1-SGR-luc contains a bicistronic replicon as follows: [JFH1-derived untranslated region (UTR; nt 1–397)]-[in frame *Renilla* luciferase reporter]-[EMCV IRES-nonstructural genes (NS3-NS5B)]. To make this plasmid, a DNA fragment encoding *Renilla* luciferase was fused between the T7 promoter sequence-5’ UTR of JFH1 and the EMCV IRES-nonstructural genes from pSGR-JFHI [60] following PCR (for oligonucleotide sequence see Table 1), digestion (with inserted *BglII* site between 5’UTR and 5’end of *Renilla*, a *PmeI* site between the 3’end of *Renilla* and 5’end of the ECMV IRES, and an existing *AgeI* site in the 5’UTR of pSGR-JFH1), and a 3-piece ligation. Mutagenesis of constructs was performed using the QuikChange lightning site-directed mutagenesis kit (Stratagene) on pJFH1-SGR-luc, pHCV-HP, psJFH1-p7+NS, pFL-J6/JFH-1-FlagE2, pEF1 NS4A-HA, or pEF-Tak Flag-E1 using the indicated oligonucleotides (Table 1). psJFH1-p7+NS ΔE1/E2 was constructed by removing amino acids 192–720 from the psJFH1 p7+NS background [24]. HCV over-expression constructs (noted below) were constructed by PCR amplification of the gene of interest from psJFH1-p7+NS and insertion of the *PmeI-NotI* digested fragment into pEF-Tak-Flag [61] or the *EcoRI-XbaI* digested fragment into pEF1. pEF-Tak Flag-NS2 was created using InFusion (Clontech) after PCR. pEF-Tak Flag-E1 (genotypes 1B and 3) and pEF1 NS4A-HA WT and Y45F (genotypes 1B and 3) were made using gBlocks (IDT) of the entire coding region with vector overlap, followed by InFusion cloning (Clontech). Table 1 provides the sequence of all oligonucleotides used. Bold letters in the oligonucleotide sequences indicate overlap with vector sequence, and the sequence of the HA tag within the oligonucleotides is underlined. All nucleotide and amino acid positions refer to the JFH1 genome (GenBank accession number: AB047639). The sequences of all plasmids were verified by DNA sequencing and are available upon request.

**Table 1:** Oligonucleotides used in this study.

Construct Name	Forward Primer (5’-3’)	Reverse Primer (5’-3’)
Oligonucleotides used for site-directed mutagenesis		
pJFH1-SGR-luc: 5’UTR to *BglII*	CATGAATCACTCCCCTGTGA	T**AGATCT**TGGGCGACGGTTGGTG
pJFH1-SGR-luc: *BglII*—*Renilla*—*PmeI*	A**AGATCT**ATGACTTCGAAAGTTTATGA TC	T**GTTTAAAC**TTATTGTTCATTTTTGAGAACTC
pJFH1-SGR-luc Y45F	CCATCTCATCAAAAGCCTCGAACAGGACCTCCTTATCCGG	CCGGATAAGGAGGTCCTGTTCGAGGCTTTTGATGAGATGG
psJFH1-p7+NS Y45F	CCATCTCATCAAAAGCCTCGAACAGGACCTCCTTATCCGG	CCGGATAAGGAGGTCCTGTTCGAGGCTTTTGATGAGATGG
pJFH1-SGR-luc K41A	CGTCGTTGCGCCGGATGCGGAGGTCCTGTATG	CATACAGGACCTCCGCATCCGGCGCAACGACG
psJFH1-p7+NS K41A	CGTCGTTGCGCCGGATGCGGAGGTCCTGTATG	CATACAGGACCTCCGCATCCGGCGCAACGACG
pJFH1-SGR-luc L44A	CGCCGGATAAGGAGGTCGCGTATGAGGCTTTTGATG	CATCAAAAGCCTCATACGCGACCTCCTTATCCGGCG
psJFH1-p7+NS L44A	CGCCGGATAAGGAGGTCGCGTATGAGGCTTTTGATG	CATCAAAAGCCTCATACGCGACCTCCTTATCCGGCG
pJFH1-SGR-luc Y45A	TTCCTCCATCTCATCAACAGCCTCATACAGGACCT	CATCTCATCAAAAGCCTCAGCCAGGACCTCCTTATCCGGC
pJFH1-SGR-luc D49A	CCTGTATGAGGCTTTTGCTGAGATGGAGGAATGCG	CGCATTCCTCCATCTCAGCAAAAGCCTCATACAGG
pJFH1-SGR-luc E52A	GCTTTTGATGAGATGGCGGAATGCGCCTCTAGG	CCTAGAGGCGCATTCCGCCATCTCATCAAAAGC
psJFH1-p7+NS E52A	GCTTTTGATGAGATGGCGGAATGCGCCTCTAGG	CCTAGAGGCGCATTCCGCCATCTCATCAAAAGC
pHCV-HP Y45F	CCCGACAGGGAAGTCCTTTTCCGGGAGTTC	GAACTCCCGGAAAAGGACTTCCCTGTCGGG
pFL-J6/JFH1-FlagE2 NS4A Y45F	CCATCTCATCAAAAGCCTCGAACAGGACCTCCTTATCCGG	CCGGATAAGGAGGTCCTGTTCGAGGCTTTTGATGAGATGG
pFL-J6/JFH1-FlagE2 E1 D72A	CTGCGGACGCACATCGCTATGGTTGTGATGTCC	GGACATCACAACCATAGCGATGTGCGTCCGCAG
pFL-J6/JFH1-FlagE2 NS4A K41A	CGTCGTTGCGCCGGATGCGGAGGTCCTGTATG	CATACAGGACCTCCGCATCCGGCGCAACGACG
pEF1 NS4A-HA Y45T	GCCGGATAAGGAGGTCCTGACTGAGGCTTTTGATGAGATG	CATCTCATCAAAAGCCTCAGTCAGGACCTCCTTATCCGGC
pEF1 NS4A-HA Y45R	GCCGGATAAGGAGGTCCTGCGTGAGGCTTTTGATGAGATG	CATCTCATCAAAAGCCTCACGCAGGACCTCCTTATCCGGC
pEF1 NS4A-HA Y45D	CGGATAAGGAGGTCCTGGATGAGGCTTTTGATGAG	CTCATCAAAAGCCTCATCCAGGACCTCCTTATCCG
pEF-Tak Flag-E1 D72A	CTGCGGACGCACATCGCTATGGTTGTGATGTCC	GGACATCACAACCATAGCGATGTGCGTCCGCAG
Oligonucleotides used for cloning into expression vectors		
pEF-Tak Flag-Core	**GATGATAAAGCGGCCGC**TAGCACAAATCCTAAACCTCAAAG	**CTGATCAGCGGGTTTAAA**CCTAAGCAGAGACCGGAACGG
pEF-Tak Flag-E1	**GATAAAGCGGCCGC**TGCCCAGGTGAAGAATAC	**CGGGTTTAAAC**CGCGTCCACCCCAGCGG
pEF-Tak Flag-E2	**ATGATAAAGCGGCCGC**TGGCACCACCACCGTT	**GGGTTTAAAC**TTCGGCCTGGCCCAACAAGA
pEF-Tak Flag-p7	**TGATAAAGCGGCCGC**TGCAGCATTGGAGAAG	**CGGGTTTAAAC**GGCATAAGCCTGCCGGG
pEF-Tak Flag-NS3	**ATAAAGCGGCCGC**TGCTCCCATCACTGCT	**CGGGTTTAAAC**GGTCATGACCTCAAGGTCA
pEF-Tak Flag-NS5A	**GATAAAGCGGCCGC**TTCCGGATCCTGGC	**CGGGTTTAAAC**GCAGCACACGGTGGTATCG
pEF NS4A-HA	**AATTCTGCAGATAGCTT**ATGAGCACGTGGGTCCT	**CTCTAGACTA**AGCGTAGTCTGGGACGTCGTATGGGTAGCATTCCTCCATC
pEF NS4A-HA Y45F	**AATTCTGCAGATAGCTT**ATGAGCACGTGGGTCCT	**CTCTAGACTA**AGCGTAGTCTGGGACGTCGTATGGGTAGCATTCCTCCATC
pEF-Tak Flag-NS2	**TGATGATGATAAAGCGGCCGC**TTATGACGCACCT	**CTGATCAGCGGGTTTAAAC**AAGGAGCTTCCACCCCT
pEF-Tak Flag-E1 1–66	**GATGATGATGATAAAGCGGCCGC**TGCCCAGGTGAAGAATA	***GGCTGATCAGCGGGTTTAAA****CCTAACCCTGCGTGAGGGCA*
pEF-Tak Flag-E1 1–106	**GATGATGATGATAAAGCGGCCGC**TGCCCAGGTGAAGAATA	***GGCTGATCAGCGGGTTTAAA****CCTAGTACTGCGGCGAGACG*
pEF-Tak Flag-E1 1–138	**GATGATGATGATAAAGCGGCCGC**TGCCCAGGTGAAGAATA	***GGCTGATCAGCGGGTTTAAA****CCTACGTGGGCGACCAGTTC*
pEF-Tak Flag-E1 67–192	**GATGATGATGATAAAGCGGCCGC**TCTGCGGACGC	**GGCTGATCAGCGGGTTTAAA**CCTACGCGTCCACCCCAGCGG
pEF-Tak Flag-E1 107–192	**GATGATGATGATAAAGCGGCCGC**TCACTGGTTTGTGCAAG	***GGCTGATCAGCGGGTTTAAA****CCTACGCGTCCACCCCAGCG*

### *In vitro* transcription of HCV RNA and electroporation

Plasmid DNA encoding the described HCV constructs was linearized using the *XbaI* restriction enzyme for full-length or SGR-luc HCV RNA or *ScaI* for HP subgenomic replicon RNA. Purified linearized DNA was used as a template for *in vitro* transcription with a MEGAscript T7 transcription kit (Thermo-Fisher). The *in vitro* transcribed RNA was treated with DNase (Thermo-Fisher) and then purified by phenol-chloroform extraction. The quality of the RNA was verified on a denaturing gel. For electroporation, 1 μg (HCV replicon RNA) or 5 μg (HCV full-length RNA) was electroporated into 4x10^6^ Huh7.5 cells in Cytomix electroporation buffer (120 mM KCl, 10 mM Potassium Phosphate Buffer, 5 mM MgCl_2_, 25 mM HEPES, 0.15 mM CaCl_2_, 2 mM EGTA, pH 7.6) at 250 V and 950 μF in a 4 mm cuvette with a Gene Pulser Xcell system (Bio-Rad). Four hours post electroporation, cells were washed extensively with Phosphate Buffered Saline (PBS) and cDMEM.

### Focus forming assay

*Extracellular titer*: Supernatants were harvested from Huh7.5 cells electroporated with HCV RNA at indicated time points, serially diluted, and used to infect naïve Huh7.5 cells in triplicate wells of a 48-well plate for 2 hours. Plates were harvested at 48 hours post infection and fixed with 4% paraformaldehyde. Cells were permeabilized (0.2% Triton-X-100 in PBS), blocked (10% FBS in PBS) and immunostained with mouse anti-HCV NS5A antibody (9e10, 1:500, gift of Dr. Charles Rice). Infected cells were visualized following incubation with horseradish peroxidase (HRP)-conjugated secondary antibody (1:500; Jackson ImmunoResearch) and VIP Peroxidase Substrate Kit (Vector Laboratories). Foci were counted at 40X magnification. Titer (FFU/mL) was determined as previously described [62]. *Intracellular titer*: Cell pellets were washed with PBS and resuspended in serum-free DMEM. Cells were then lysed using a series of freeze/thaw cycles in a dry ice/Ethanol bath. Post-nuclear supernatants were used to infect naïve Huh7.5 cells, and a focus forming assay was performed as described above.

### Luciferase assays

JFH1 SGR-luc *in vitro* transcribed RNA (1 μg) was electroporated into Huh7.5 cells. Cells were suspended in 20 mL cDMEM and plated in 12-well plates. Cells were harvested after a PBS wash by incubation in *Renilla* lysis buffer (Promega). *Renilla* luciferase values were measured according to manufacturer’s instructions (Renilla Luciferase Assay System, Promega) using a BioTek Synergy 2 microplate reader.

### HCV NS4A sequencing

RNA was extracted from cells by using the Qiagen RNeasy kit according to manufacturer’s instructions and then used as a template for cDNA synthesis with the Superscript cDNA synthesis kit (Thermo Fisher). The NS4A region of the HCV genome was amplified by nested PCR with the following oligonucleotides: Round 1: 5’–CAGTCCGATGGAGAAGAAGG—3’, 5’—GCATGGGATGGGGCAGTC—3’, Round 2: 5’—ACACATAGACGCCCACTTCC—3’, 5’—GTATGTCCTGGGCCTGCTTA—3’, and then the 542 bp PCR product was purified and sequenced by Sanger sequencing.

### Quantification of HCV RNA by RT-qPCR

RNA from cells was isolated using the RNeasy kit (Qiagen), and RNA from infected supernatants was isolated using the QIAamp viral RNA kit (Qiagen), both according to manufacturer’s instructions. The RNA copy number of harvested RNA was measured in triplicate by RT-qPCR using the TaqMan Fast Virus 1-Step Mix (Qiagen) with an HCV-specific probe targeting the 5’ untranslated region of HCV (Assay ID: Pa03453408_s1). The copy number was calculated by comparison to a standard curve of a full-length *in vitro* transcribed HCV RNA, as described [58].

### Immunoblotting

Cells were lysed in a modified radio immunoprecipitation assay (RIPA) buffer (10 mM Tris pH 7.5, 150 mM NaCl, 0.5% sodium deoxycholate, 1% Triton X-100) supplemented with protease inhibitor cocktail (Sigma) and phosphatase inhibitor cocktail (Millipore), and post-nuclear supernatants were harvested by centrifugation. Quantified protein was resolved by SDS/PAGE, transferred to PVDF membranes using the Turbo-transfer system (BioRad) and blocked with StartingBlock (Thermo-Fisher) or 3% bovine serum albumin (Sigma) in PBS with 0.1% Tween (PBS-T). Membranes were probed with specific antibodies, washed with PBS-T and incubated with species-specific HRP conjugated antibodies (Jackson ImmunoResearch, 1:5000), washed again with PBS-T, and treated with Pico PLUS enhanced chemiluminescent (ECL) reagent (Thermo-Fisher). The signal was then captured on X-ray film or by using a LICOR Odyssey FC. Antibodies used for immunoblot include mouse anti-HCV Core (1:250, Abcam), mouse anti-HCV NS3 (1:500, Abcam), rabbit anti-HCV NS4A (1:1000, Genscript [63]), mouse anti-HCV NS5A (1:500, 9e10, gift of Dr. Charles Rice), anti-Flag HRP (1:2500, Sigma), rabbit anti-HA (1:500, Sigma), and mouse anti-E1 (1:1000, A4, gift of Dr. Charles Rice).

### Proteolytic protection assay

This protocol was adapted from the manuscript by Gentzsch and colleagues [24]. Briefly, cells electroporated with JFH1-p7+NS *in vitro* transcribed RNA were harvested at 48 hours post electroporation by scraping into cold proteinase K buffer (50 mM Tris-HCl pH 8.0, 10 mM CaCl_2_, 1 mM DTT). Cells were then lysed by five freeze/thaw cycles and aliquots of lysate (50 μL) were either (i) left untreated, (ii) pretreated with 5 μL of 10% Triton-X-100 followed by proteinase K treatment (50 μg/mL) for 30 minutes on ice, or (iii) treated with proteinase K only. Proteinase K treatment was terminated by incubation with 10 mM phenylmethane sulfonyl fluoride. The samples were mixed with 4X SDS sample buffer (1 M Tris (pH 6.8), 60% glycerol, 0.06% Bromophenol Blue, 12% SDS)), incubated at 50°C for 5 minutes, and immunoblotted for HCV Core protein, as above.

### Immunoprecipitations

300–500 μg of protein extracted as above was incubated with 50 μL anti-Flag M2 magnetic beads (Sigma) in 1X Tris buffered saline (TBS) at 4°C overnight with head over tail rotation. Beads were washed 3X in modified 1X RIPA buffer and eluted in 2X Laemmli Buffer (BioRad) at 50°C for 5 minutes. Protein was resolved by SDS/PAGE and immunoblotting, as above. In the anti-HA immunoprecipitations, protein was incubated with equivalent amounts of anti-HA or IgG antibodies in 1X TBS at 4°C overnight with head over tail rotation. Then Protein G Dynabeads (Thermo-Fisher) were added and rotated at 4°C for two hours. Beads were washed and eluted as above.

### HCV replicon transduction assay

*In vitro* transcribed genotype 1B HP-HCV RNA (linearized by *ScaI*) was electroporated as above into Huh7.5 cells and plated into 10 cm plates at 2*10^4^ or 2*10^3^ cells per plate, along with cells electroporated with a non-replicating control. Cells were washed thoroughly with 1X PBS and cDMEM at 4 hours post electroporation. cDMEM containing 0.4 mg/mL G418 (Life Technologies) was added to cells at 24 hours post electroporation to begin selection. Cells were fixed after 3 weeks under G418 selection and stained with crystal violet (Sigma) in 20% methanol. Colonies from triplicate plates were counted and each normalized to the average number of colonies on WT plates.

### ELISA for HCV Core protein

Core protein was quantified from filtered supernatants from Huh7.5 cells 72 hours post-electroporation using the HCV Core Antigen ELISA kit according to the manufacturer’s instructions (Cell Biolabs).

### Biochemical subcellular fractionation

Concentrated supernatants were purified over a 10–50% iodixanol gradient, as previously described [50]. Briefly, at 48 hours post electroporation of HCV RNA in Huh7.5 cells, supernatant was collected, mixed with polyethylene glycol (PEG) 8000 to a final concentration of 8%, and incubated with rocking at 4°C overnight. PEG supernatants were centrifuged at 11,000 X g for 30 minutes, supernatant was removed, and remaining pellets were suspended in cold 1X PBS. These resuspensions were layered over a 10–50% iodixanol gradient and centrifuged at 222,000 X g for 16 hours in a SW41 rotor in a Beckman Coulter ultracentrifuge. 10 equal fractions (1 ml) were collected with a BioComp piston gradient fractionator, and then viral titer (FFU/ml), HCV RNA copy number (RT-qPCR), and Core protein (immunoblotting) were measured from each fraction. In some experiments (indicated in the figure legend), the protein from the gradients was incubated for 30 minutes at 37°C in 2X Urea Loading Buffer (50 mM Tris-HCL, 1.6% SDS, 7% glycerol, 8 M Urea, 4% 2-mercaptoethanol, Bromophenol Blue) with vortexing every 10 minutes. It was then boiled at 95°C for 10 minutes prior to SDS-PAGE and immunoblotting.

### Statistical analysis

Student’s unpaired t-tests and one-way analysis of variance (ANOVA) were used for statistical analysis of data. Values are presented as mean ± standard error of the mean for biological replicates or standard deviation for technical replicates (n = 3, or as indicated). *—P < 0.05, **—P < 0.01, ***—P < 0.001, ****—P < 0.0001.

## Supporting information

S1 Fig Role of NS4A Y45 in HCV replication and E1 interaction in multiple genotypes. (A) Representative images of Huh7.5 cells electroporated with either WT or Y45F *in vitro* transcribed genotype 1B HP subgenomic replicon RNA. Cells were plated in serial dilutions as indicated and then stained with crystal violet after three weeks of G418 selection. (B) Quantification of colony numbers from A. Data is presented as mean ± SEM (n = 3). (C-D) Immunoblot analysis of anti-Flag immunoprecipitated extracts or whole cell lysate (WCL) from Huh7.5 cells transfected with indicated HCV proteins from either genotype 1B (C) or genotype 3 (D) or vector. Panels are representative of three independent experiments.
(TIF)

S2 Fig NS4A binds to Core and NS5A but not p7 or NS2. Immunoblot analysis of anti-Flag immunoprecipitated extracts and whole cell lysate (WCL) from Huh7.5 cells transfected with NS4A-HA WT, NS4A-HA Y45F, and vector or Flag-tagged Core (A), E2 (B), p7/NS2 (C), or NS5A (D).
(TIF)

S3 Fig NS4A Y45T and Y45D, but not Y45R, bind to the E1 glycoprotein. Immunoblot analysis of anti-Flag immunoprecipitated extracts and whole cell lysate (WCL) from Huh7.5 cells transfected with the indicated HA-NS4A proteins and Flag-tagged E1 or vector.
(TIF)

S4 Fig Supernatant composition of NS4A K41A differs from that of E1 D72A and NS4A Y45F. Supernatants from Huh7.5 cells electroporated with *in vitro* transcribed WT, NS4A Y45F, E1 D263A, or NS4A K41A *in vitro* transcribed RNA were concentrated, fractionated over a 10–50% iodixanol gradient, and collected in 10 equal fractions. Fractions were analyzed by focus-forming assay for infectivity and RT-qPCR for HCV RNA (A) and fractions 3 and 4 were analyzed for HCV structural proteins by immunoblot (B). Fractions from left to right correspond with fractions running from top to bottom of the gradient, and the density of each is listed below. Data in A is presented as mean ± SD (n = 3), A and B are representative of 2 independent experiments.
(TIF)
