# Supplementary figures and images for: The acidic domain of the hepatitis C virus NS4A protein is required for viral assembly and envelopment through interactions with the viral E1 glycoprotein
Source: PLoS Pathog. 2019 Feb 7;15(2):e1007163. doi: 10.1371/journal.ppat.1007163 (PMC6382253; doi:10.1371/journal.ppat.1007163)

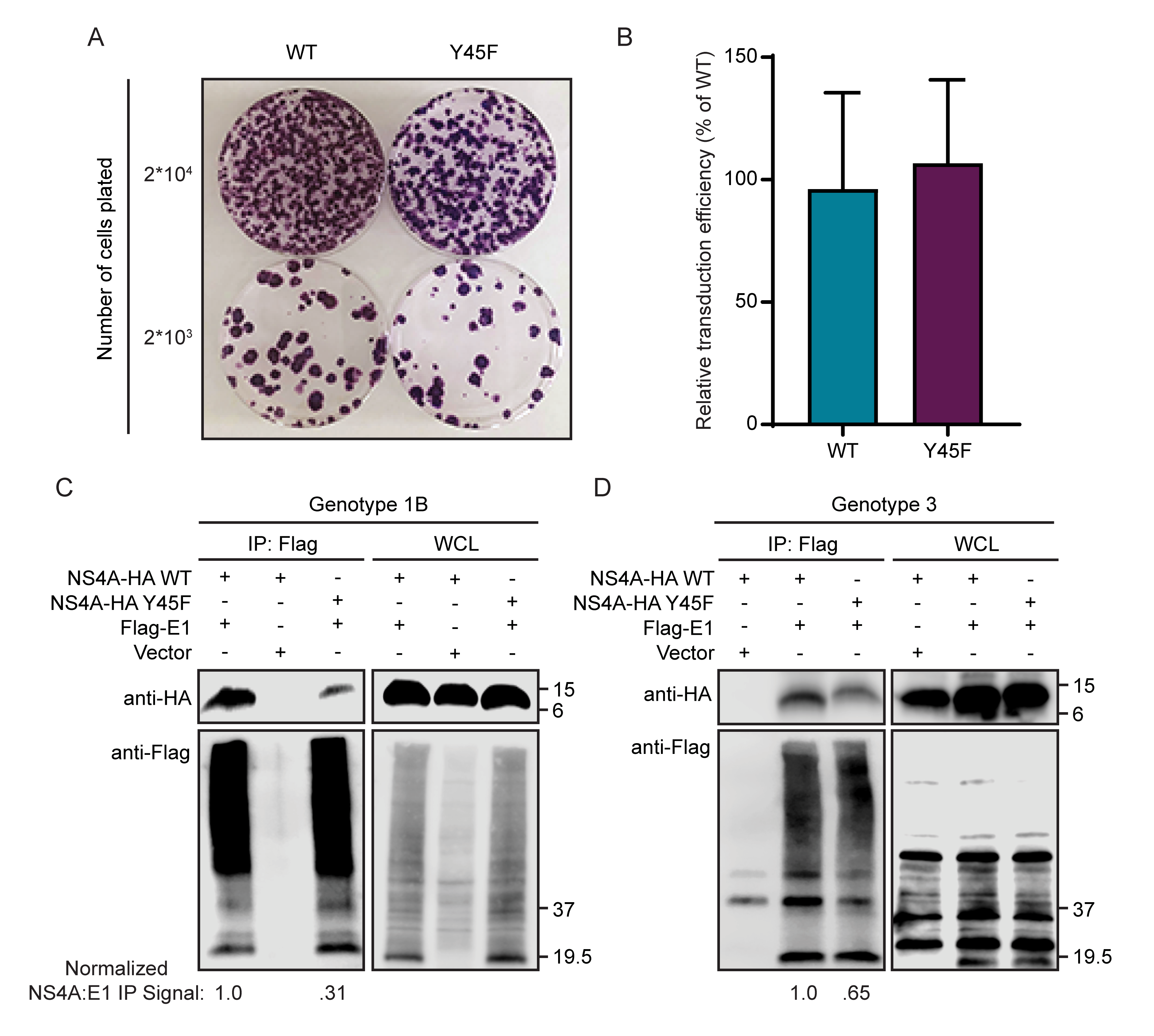

Supplement: S1 Fig — (A) Representative images of Huh7.5 cells electroporated with either WT or Y45F in vitro transcribed genotype 1B HP subgenomic replicon RNA. Cells were plated in serial dilutions as indicated and then stained with crystal violet after three weeks of G418 selection. (B) Quantification of colony numbers from A. Data is presented as mean ± SEM (n = 3). (C-D) Immunoblot analysis of anti-Flag immunoprecipitated extracts or whole cell lysate (WCL) from Huh7.5 cells transfected with indicated HCV proteins from either genotype 1B (C) or genotype 3 (D) or vector. Panels are representative of three independent experiments. (TIF) [file ppat.1007163.s001.tif]

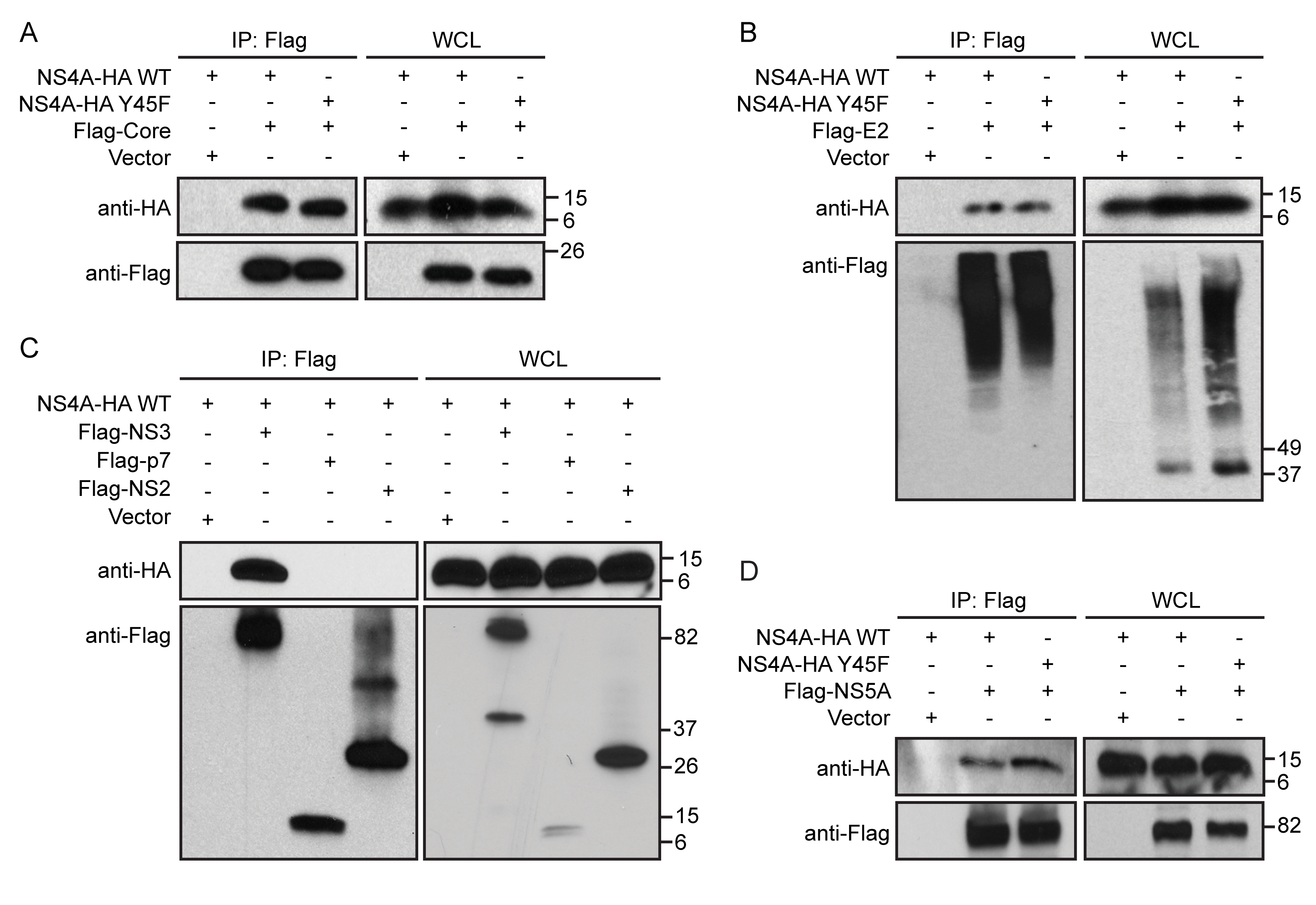

Supplement: S2 Fig — Immunoblot analysis of anti-Flag immunoprecipitated extracts and whole cell lysate (WCL) from Huh7.5 cells transfected with NS4A-HA WT, NS4A-HA Y45F, and vector or Flag-tagged Core (A), E2 (B), p7/NS2 (C), or NS5A (D). (TIF) [file ppat.1007163.s002.tif]

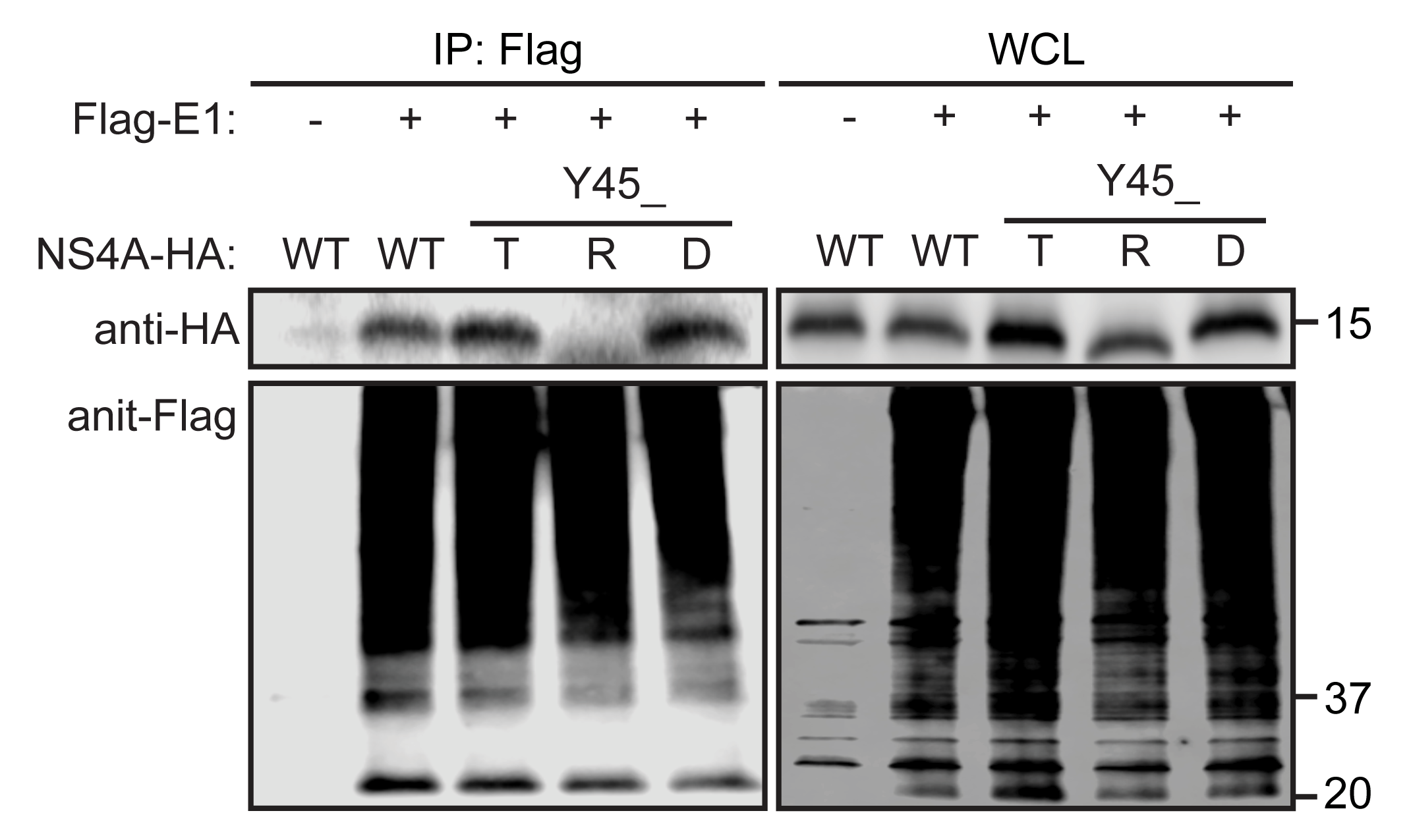

Supplement: S3 Fig — Immunoblot analysis of anti-Flag immunoprecipitated extracts and whole cell lysate (WCL) from Huh7.5 cells transfected with the indicated HA-NS4A proteins and Flag-tagged E1 or vector. (TIF) [file ppat.1007163.s003.tif]

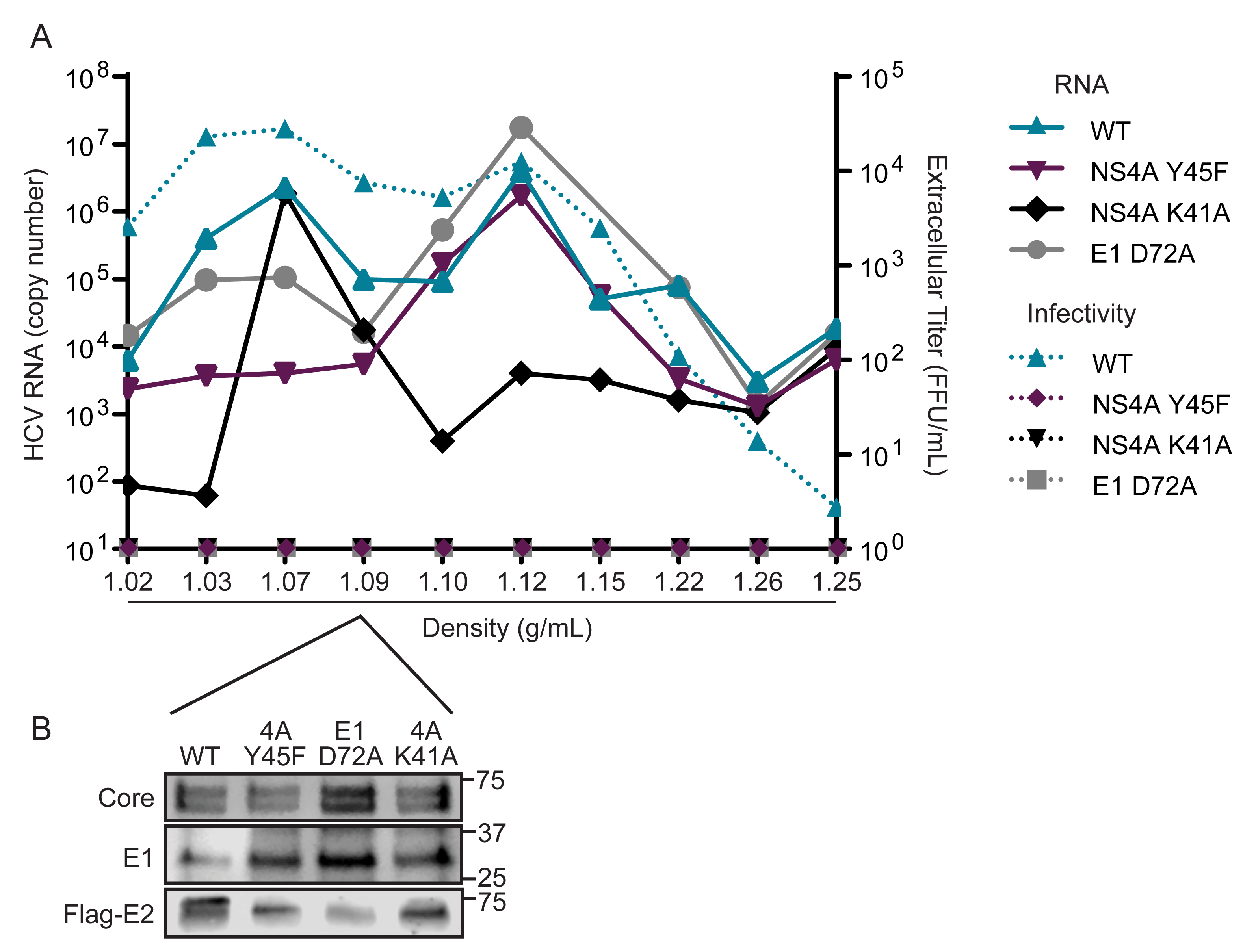

Supplement: S4 Fig — Supernatants from Huh7.5 cells electroporated with in vitro transcribed WT, NS4A Y45F, E1 D263A, or NS4A K41A in vitro transcribed RNA were concentrated, fractionated over a 10–50% iodixanol gradient, and collected in 10 equal fractions. Fractions were analyzed by focus-forming assay for infectivity and RT-qPCR for HCV RNA (A) and fractions 3 and 4 were analyzed for HCV structural proteins by immunoblot (B). Fractions from left to right correspond with fractions running from top to bottom of the gradient, and the density of each is listed below. Data in A is presented as mean ± SD (n = 3), A and B are representative of 2 independent experiments. (TIF) [file ppat.1007163.s004.tif]
